# Clinical and preclinical evaluation of miR‐144‐5p as a key target for major depressive disorder

**DOI:** 10.1111/cns.14291

**Published:** 2023-06-12

**Authors:** Xiaodong Wu, Yulong Zhang, Ping Wang, Xiaohui Li, Zhen Song, Chuke Wei, Qing Zhang, Bei Luo, Zhichun Liu, Yingying Yang, Zhenhua Ren, Huanzhong Liu

**Affiliations:** ^1^ Department of Psychiatry Chaohu Hospital of Anhui Medical University Hefei China; ^2^ Department of Psychiatry, School of Mental Health and Psychological Sciences Anhui Medical University Hefei China; ^3^ Department of Psychiatry, Anhui Psychiatric Center Anhui Medical University Hefei China; ^4^ Department of Anatomy Anhui Medical University Hefei China

**Keywords:** depression, miR‐144‐5p, neuroinflammation, neuronal destruction

## Abstract

**Background:**

Neuronal abnormalities are closely associated with major depressive disorder (MDD). Available evidence suggests a role for microRNAs (miRNAs) in regulating the expression of genes involved in MDD. Hence, miRNAs that can be potential therapeutic targets need to be identified.

**Methods:**

A mouse model of chronic unpredictable stress (CUS) was used to evaluate the function of miRNAs in MDD. miR‐144‐5p was screened from the hippocampi of CUS mice based on sequencing results. Adenovirus‐associated vectors were used to overexpress or knockdown miR‐144‐5p in mice. BpV(pic) and LY294002 were used to determine the relationship between miR‐144‐5p target genes PTEN and TLR4 in neuronal impairment caused by miR‐144‐5p deficiency. Western blotting, immunofluorescence, ELISA immunosorbent assay, and Golgi staining were used to detect neuronal abnormalities. Serum samples from healthy individuals and patients with MDD were used to detect miR‐144‐5p levels in the serum and serum exosomes using qRT‐PCR.

**Results:**

miR‐144‐5p expression was significantly decreased within the hippocampal dentate gyrus (DG) of CUS mice. Upregulation of miR‐144‐5p in the DG ameliorated depression‐like behavior in CUS mice and attenuated neuronal abnormalities by directly targeting PTEN and TLR4 expression. Furthermore, miR‐144‐5p knockdown in normal mice led to depression‐like behavior via inducing neuronal abnormalities, including abnormal neurogenesis, neuronal apoptosis, altered synaptic plasticity, and neuroinflammation. miR‐144‐5p deficiency‐mediated neuronal impairment was mediated by PI3K/Akt/FoxO1 signaling. Furthermore, miR‐144‐5p levels were downregulated in the sera of patients with MDD and associated with depressive symptoms. Consistently, serum exosome‐derived miR‐144‐5p levels were decreased in patients with MDD.

**Conclusion:**

miR‐144‐5p plays a vital role in regulating neuronal abnormalities in depression. Our findings provide translational evidence that miR‐144‐5p is a new potential therapeutic target for MDD.

## Graphical abstract

Schematic illustration of miR‐144‐5p ameliorating neuronal abnormalities caused by chronic stress.

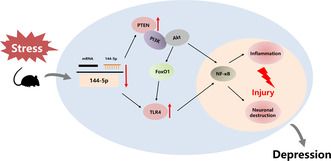

## 1 INTRODUCTION

Depression, which is characterized by mood disorders, is one of the most common mental illnesses, affecting approximately 322 million people worldwide.^1^ Genetic and environmental factors contribute to the etiology of major depressive disorder (MDD).^2^ Stress is a significant risk factor for the onset of depression.^3^ Owing to the absence of clinically specific biomarkers, MDD is diagnosed mainly based on subjective symptoms. No more than one‐third of patients achieve favorable outcomes after antidepressant therapy.^4^ Neuroinflammation is believed to be involved in the pathophysiology of depression.^5^, ^6^, ^7^ Increased levels of inflammatory factors such as IL‐1β, IL‐6, and TNF‐α are associated with depression‐like behavior.^8^, ^9^ The hippocampus is a part of the limbic system and plays a crucial role in the pathophysiology of depression.^10^, ^11^, ^12^, ^13^ Exposure of mice to chronic stress leads to increased neurodegeneration in the hippocampus.^14^ Patients with depression show reduced hippocampal volume,^15^ and this shrinkage is positively correlated with the duration of MDD.^16^, ^17^ A previous study revealed that depression‐like behaviors were accompanied by inflammation and neuronal destructionvia mechanisms, including apoptosis.^18^ Hence, identification of potential therapeutic targets for MDD is needed.

MicroRNAs (miRNAs) are small non‐coding RNAs that modify protein translation to regulate gene expression.^19^ Dysfunction of specific miRNAs contributes to neuropsychiatric diseases,^20^, ^21^, ^22^ including MDD.^23^Diverse miRNA changes in the ventral prefrontal cortex, serum, and cerebrospinal fluid of patients with MDD suggest that miRNAs are involved in MDD pathogenesis.^22^ Hence, specific downstream miRNAs associated with depression pathogenesis should be investigated.

In the present study, we investigated different miRNAs in a mouse model of chronic unpredictable stress (CUS)‐induced depression and explored the potential molecular mechanisms by which these miRNAs mediate neuronal abnormalities in the hippocampal dentate gyrus (DG). Furthermore, we validated the selected miRNA in peripheral serum samples from patients with MDD. Our results provide evidence that miRNAs can be new therapeutic targets for depression.

## 2 MATERIALS AND METHODS

### 2.1 Participants

Patients with MDD (age: 18–60 years) were enrolled in the study. The Ethics Committee at Chaohu Hospital of Anhui Medical University approved the study protocol (approval ID: KYXM‐202112‐010). The 24‐item Hamilton Depression Rating Scale (HAMD‐24) and Hamilton Anxiety Scale (HAMA) were utilized to evaluate MDD severity. Patients with first‐episode depression without any medication or off medication for over 3 weeks, and only depressed candidates with HAMD scores ≥20 were recruited. Pregnant patients and those with a primary diagnosis of schizophrenia or other psychotic disorders, substance abuse, severe cognitive impairment, or epilepsy were excluded from the study. Patients with other mental illnesses or cognitive impairments were also excluded. Individuals with no history of mental illness were included in the healthy control (HC) group. Informed consent was obtained from all participants. This study comprised 48 individuals, 24 with MDD and 24 healthy individuals.

### 2.2 Animals and CUS model

Male C57BL6/J mice (license number: SCXK Wan 2,019,007), 6–8 weeks old, were purchased from the Animal Experiment Center of Anhui Medical University (Hefei, China). All mice were maintained under a 12‐h light/dark cycle. All procedures were conducted in compliance with the ARRIVE guidelines. The CUS model was established as previously described, with minor modifications.^24^ Mice were subjected to different, randomized, low‐intensity stressors, with 2–3 stressors per day, for 5 weeks. The following stress stimuli were used: overnight illumination, 24 h water or food deprivation, 5 min swimming (at 4–8°C or 28–35°C), 6 h physical restrain, 45° cage tilt, and 5 min tail nipping. In total, 268 mice were used in this study.

### 2.3 Behavioral assessment

Behavioral assessments were performed in a quiet test room by two researchers who were blinded to the animal groups. AZH‐SBS Animal Behavior Video Analysis System was used to analyze the behavioral test data.

The sucrose preference test (SPT) was performed as previously described^25^ using the formula: (sucroseintake/total intake) ×100%. During the adaptation stage, bottles of 1% sucrose water and pure water were placed in each cage (single‐cage rearing). The positions of the two bottles were reversed every 12 h to eliminate the influence of position preference on results. During testing, the mice were deprived of food and water for the first 24 h and allowed free access to the two bottles for 6 h. The sucrose preference was calculated using the following formula: (sucrose intake/total intake) ×100%.

The tail suspension test (TST) was performed as previously described with minor modifications.^26^ The mice were taped approximately 1 cm from the tail tip and suspended head down using a plastic rod. A high‐definition camera recorded behavior during a 6‐min test session. Immobility duration was measured in the last 4 min after the 2‐min adaptation period. Complete limb immobility was defined as immobility.

Forced swim test (FST) was performed as previously described.^26^ The mice were placed in a water‐filled cylinder (depth: 15 cm × height: 30 cm × diameter: 25 cm). A high‐definition camera was used to record the mice swimming for 6 min and to determine the immobility period during the last 4 min. Immobility was defined as the absence of any motion other than that which kept the head above the water.

Open‐field test (OFT) was performed as previouslydescribed^27^ to detect locomotor activity and exploration. Mice were laid in the field (50 × 50 × 40 cm) for 2 min to acclimatize to the environment, and activities were recorded for the next 3 min. The total distance was recorded.

### 2.4 miRNA sequencing and luciferase reporter assay

RNA sequencing was outsourced to OeBiotech Co., Ltd. (Shanghai, China). Briefly, total RNA was isolated from hippocampal tissues using TRIzol reagent (Invitrogen, USA) and amplified using the polymerase chain reaction. The amplicons were sequenced on an Illumina HiSeq 2500 platform. Diana miRpath was used to analyze differentially expressed miRNAs and *p* < 0.05 was defined as the threshold for significant differences. Significant correlations between the miRNA‐mediated signaling pathways were investigated using the KEGG database. The target genes of miR‐144‐5p were predicted based on the TargetScan and miRWalk databases, and a dual‐luciferase assay was performed to assess whether it directly binds to PTEN and TLR4. Wild‐type (WT) and mutant type (MUT) reporter plasmids targeting PTEN and TLR4 expression were synthesized. WT and MUT were individually co‐transfected with the miR‐l44‐5p‐mimic or NC‐mimic into HEK‐293 T cells for 48 h and assayed for firefly luciferase activity.

### 2.5 Adenovirus‐associated vector (AAV) and drug administration

Anesthesia was induced using sodium pentobarbital (45 mg/kg, intraperitoneally). AAV9‐CMV‐eGFP‐Sponge (miR‐144‐5p)‐WPRE vector (AAV‐miR‐144‐5p sponge) and AAV9‐CMV‐eGFP‐miR‐144‐5p vector (AAV‐miR‐144‐5p) (GeneChem, Shanghai, China) were used to regulate endogenous miR‐144‐5p expression. We injected AAV (~1 × 10^12^ infection units per mL, 1 μL) into the DG region bilaterally based on coordinates of the Mouse Brain Atlas (AP, −1.9 mm; ML, ±1.1 mm; DV, −2.0 mm). BpV(pic) (Sigma, SML0885) was dissolved in 1% DMSO and intraperitoneally injected (0.2 mg/kg) as previously described.^28^ LY294002 (MCE, HY‐10108, 6 μg) was administered intranasally 1 h before bpV(pic) treatment.^29^

### 2.6 Preparation and identification of serum exosomes

Serum samples were mixed with the ExoQuick Exosome Precipitation Solution (EXOQ20A‐1, System Biosciences) according to the manufacturer's instructions. Exosomal features were confirmed using marker proteins CD9 (20597‐1‐AP, Proteintech Group) and CD63 (YT5525, Immunoway), transmission electron microscopy (TEM, Thermo Scientific Talos L120C G2, USA), and nanoparticle tracking analysis (NTA, Nanosight NS300, UK).

### 2.7 Real‐time quantitative PCR (qRT‐PCR)

TRIzol reagent was used to extract total RNA, followed by reverse‐transcription using a first‐strand cDNA synthesis kit. Finally, total miRNA was quantified using the miRNA qRT‐PCR detection kit (Sparkjade, China) according to the manufacturer's instructions.

### 2.8 Western blotting

A previously published procedure was followed with minor modifications.^30^ Under the stereomicroscope, hippocampal tissue was separated from DG and CA1 regions along the bisected hippocampal fissure on the ventral surface. Tissue samples from the DG were homogenized in ice‐cold lysis buffer containing protease inhibitors. Proteins were extracted using RIPA lysis buffer (G2002, Servicebio) and separated by performing SDS‐PAGE using 8%–12% resolving gels. The resolved proteins were electroblotted onto membranes. After blocking with 5% skim milk, membranes were incubated with primary antibodies listed in Table S1. ImageJ software was used to quantify the immunoreactive bands.

### 2.9 Golgi staining

The brains were swiftly removed and immersed in a Golgi staining solution. After 2 days, the staining solution was replaced with fresh staining solution and subsequently, the spent solution was replaced with fresh solution every 3 days for 14 days. The samples were cut into 100‐micron sections using a vibrating microtome, washed with xylene, air‐dried, and mounted with neutral balsam and cover glasses. Morphology of dendritic spines was assessed using light microscopy, and spine density was quantified using ImageJ software.

### 2.10 ELISA

The concentrations of TNF‐α, IL‐6, and IL‐1β in murine hippocampal DG were detected using the ELISA Kit according to the manufacturer's instructions (Table S1).

### 2.11 Immunofluorescence (IF)

IF was performed as previously described.^31^ Briefly, mice were perfused with 100 mL PBS and 25 mL 4% paraformaldehyde, and the brain tissue was isolated and sectioned to 30 μm thickness. The sections were permeabilized with Triton X‐100(0.3%) for 30 min and blocked with goat serum (3%) for 2 h. The frozen coronal slices were incubated with the primary antibodies overnight at 4°C (Table S1), followed by incubation with Cy3‐conjugated goat anti‐rabbit IgG (H+L) and Cy5‐conjugated goat anti‐mouse IgG (H+L). Images were captured using a confocal microscope (TCSSP5; Leica, Germany).

### 2.12 Statistical analysis

Results were analyzed using GraphPad Prism 9.3, in which data are expressed as the mean ± SEM. The Shapiro–Wilk test was used to determine data distribution. Student's *t*‐test or analysis of variance (ANOVA) was used for normally distributed variables. Variables that did not exhibit a normal distribution were analyzed using the Mann–Whitney or Kruskal–Wallis test. Pearson's correlation coefficient was used to measure correlation. Statistical significance was set at *p* < 0.05.

## 3 RESULTS

### 3.1 miR‐144‐5p is downregulated in CUS mice

**FIGURE 1:**
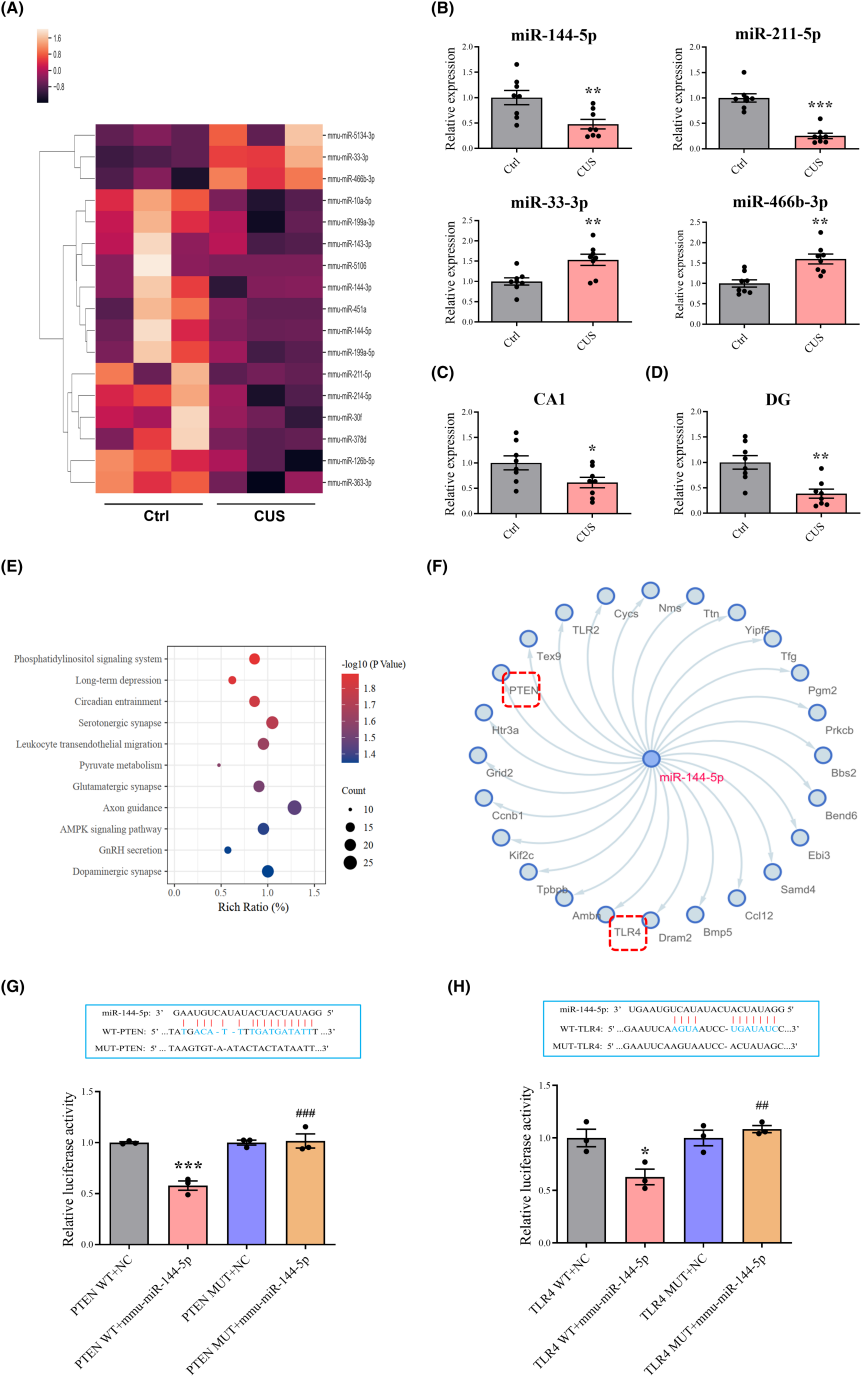
miR‐144‐5p is downregulated in CUS mice and validation of miR‐144‐5p target genes. (A) Heatmap of differentially expressed miRNAs in the hippocampus of CUS mice compared with the control group. *n* = 3 in each group. Fold change ≥1.5, *p* < 0.05. (B) The levels of miR‐144‐5p, miR‐211‐5p, miR‐33‐3p, and miR‐466b‐3p in the hippocampus. *n* = 8 in each group. The levels of miR‐144‐5p in CA1 (C) and DG (D). *n* = 8 in each group. *p < 0.05, **p < 0.01, ***p < 0.001 versus Ctrl. (E) Enrichment analysis of miR‐144‐5p target genes with KEGG. (F) Bioinformatical prediction of miR‐144‐5p target genes. (G,H) Results of putative seed‐matching sites and luciferase reporter assays verified on 293 T cells. *n* = 3 in each group. *p < 0.05, ***p < 0.001 versus WT+NC, ^##^ *p* < 0.01, ^###^ *p* < 0.001 versus WT+ mmu‐miR‐144‐5p.

To determine the potential involvement of miRNAs in CUS‐induced depression, high‐throughput sequencing was performed to identify the miRNA expression profiles in the hippocampus (Figure 1A; Table S2). Differentially expressed miRNAs, including miR‐211‐5p, miR‐144‐5p, miR‐33‐3p, and miR‐466b‐3p, were validated using qRT‐PCR (Figure 1B).The levels of miR‐144‐5p (CA1: *p* < 0.05, DG: *p* < 0.01) were significantly reduced in CUS mice (Figure 1C,D). Bioinformatics analysis results showed that among these miRNAs, miR‐144‐5p is involved in long‐term depression (LTD) and plays a vital role in synaptic plasticity (Figure 1E). Moreover, synaptic plasticity is an important pathological mechanism in the development of depression.^32^ Analysis based on TargetScan and miRWalk databases revealed that miR‐144‐5p may regulate PTEN and TLR4 as its target genes (Figure 1F). Dual‐luciferase reporter assay verified that both PTEN (Figure 1G) and TLR4 (Figure 1H) were directly targeted by miR‐144‐5p.

### 3.2 miR‐144‐5p restoration protects against behavioral dysfunctions in CUS mice

**FIGURE 2:**
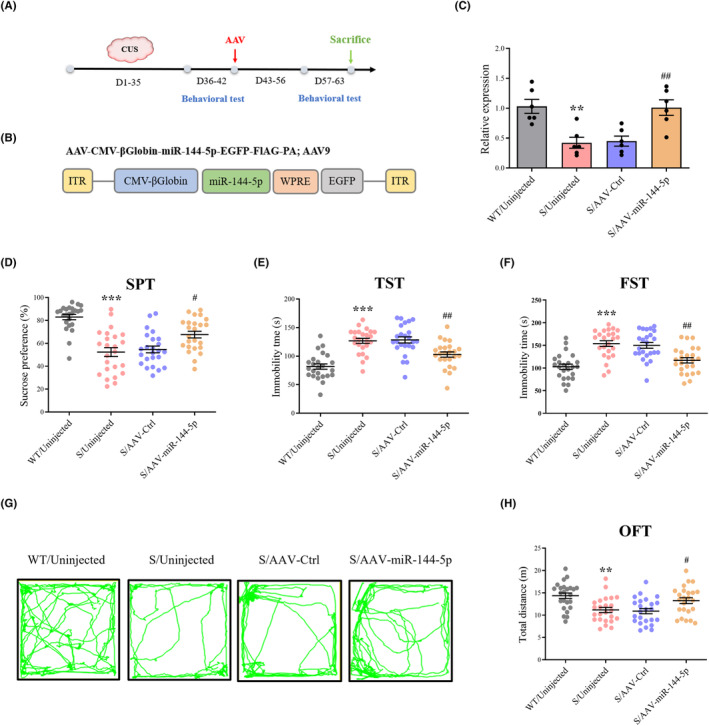
Upregulation of miR‐144‐5p rescues depression‐like phenotypes in CUS mice. (A) Experimental paradigm for AAV‐miR‐144‐5p intervention. (B) Schematics of AAV‐miR‐144‐5p. (C) The infection efficiency of AAV‐miR‐144‐5p. *n* = 6 in each group. **p < 0.01 versus WT/Uninjected group, ^##^ *p* < 0.01 versus S/AAV‐Ctrl group. (D–H) Behavioral effects of expressed AAV‐miR‐144‐5p in the DG. Upregulation of miR‐144‐5p relieved depressive‐like phenotypes in CUS mice as measured by the SPT (D), TST (E), FST (F), and OFT (G,H). *n* = 24 in each group. **p < 0.01, ***p < 0.001 versus WT/Uninjected group, ^#^ *p* < 0.05, ^##^ *p* < 0.01 versus S/AAV‐Ctrl group.

Figure 2A shows the timeline of the experimental procedures. The AAV system was employed to overexpress miR‐144‐5p and explore its potential mechanism in CUS mice (Figure 2B). Significant upregulation of miR‐144‐5p was observed after AAV injection (Figure 2C). CUS mice exhibited a range of behavioral impairments, including reduced sucrose intake in the SPT (Figure 2D), increased immobility period in the TST (Figure 2E) and FST (Figure 2F), and decreased total distance covered in the OFT (Figure 2G,H). Figure 2G shows representative search strategies in the OFT. These effects were substantially reversed in CUS mice overexpressing miR‐144‐5p (Figure 2D–H).

### 3.3 miR‐144‐5p restoration suppresses TLR4 and PTEN expression and increases cell proliferation and neurogenesis in CUS mice

**FIGURE 3:**
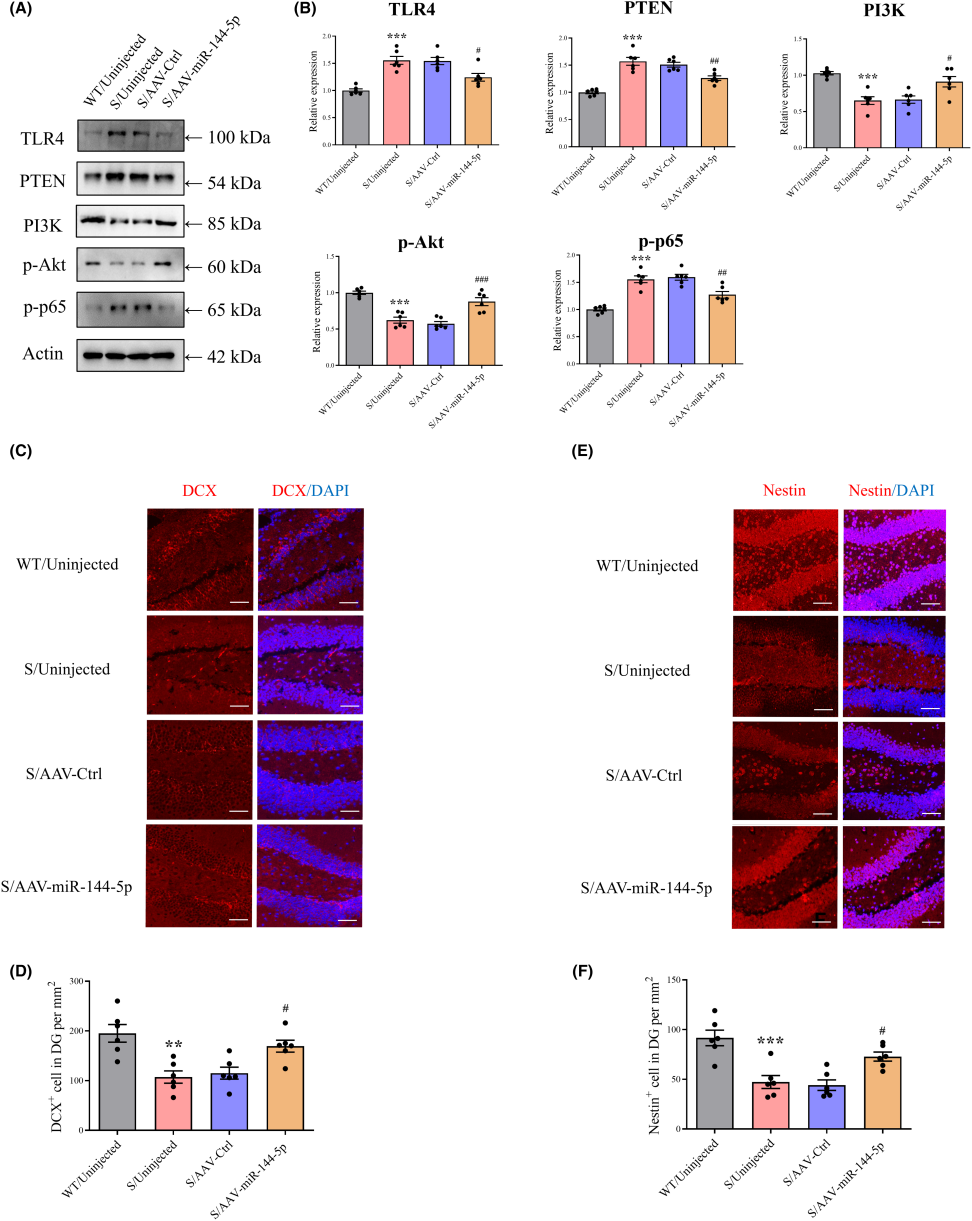
Upregulation of miR‐144‐5p promotes neuronal neurogenesis in the DG. (A) Representative bands acquired from an experiment of TLR4, PTEN, PI3K, p‐Akt, and p‐p65. (B) Overexpression of miR‐144‐5p reduced levels of TLR4, PTEN, and p‐p65 while reversing decrease in PI3K and p‐Akt induced by CUS. *n* = 6 in each group. ***p < 0.001 verus WT/Uninjected group, ^#^ *p* < 0.05, ^##^ *p* < 0.01, ^###^ *p* < 0.001 versus S/AAV‐Ctrl group. (C–F) Representative photomicrographs showing and DCX^+^ and Nestin^+^ cells in the DG. Scale bar: 50 μm. *n* = 6 in each group. **p < 0.01, ***p < 0.001 verus WT/Uninjected group, ^#^ *p* < 0.05 versus S/AAV‐Ctrl group.

The levels of PTEN, TLR4, NF‐κB, and p‐p65 were significantly elevated in CUS mice, along with decreased expression of PI3K and p‐Akt, compared with normal mice (Figure 3A,B). miR‐144‐5p upregulation inhibited PTEN and TLR4 expression. The intervention also markedly reversed the downregulation of PI3K and p‐Akt and the upregulation of NF‐κB p‐p65. IF was performed to investigate the effects of miR‐144‐5p on neurogenesis. Interestingly, upregulation of miR‐144‐5p rescued the decrease in DCX and nestin levels in CUS mice (Figure 3C–F).

### 3.4 miR‐144‐5p restoration ameliorates CUS‐induced neuronal damage and synaptic plasticity impairment

**FIGURE 4:**
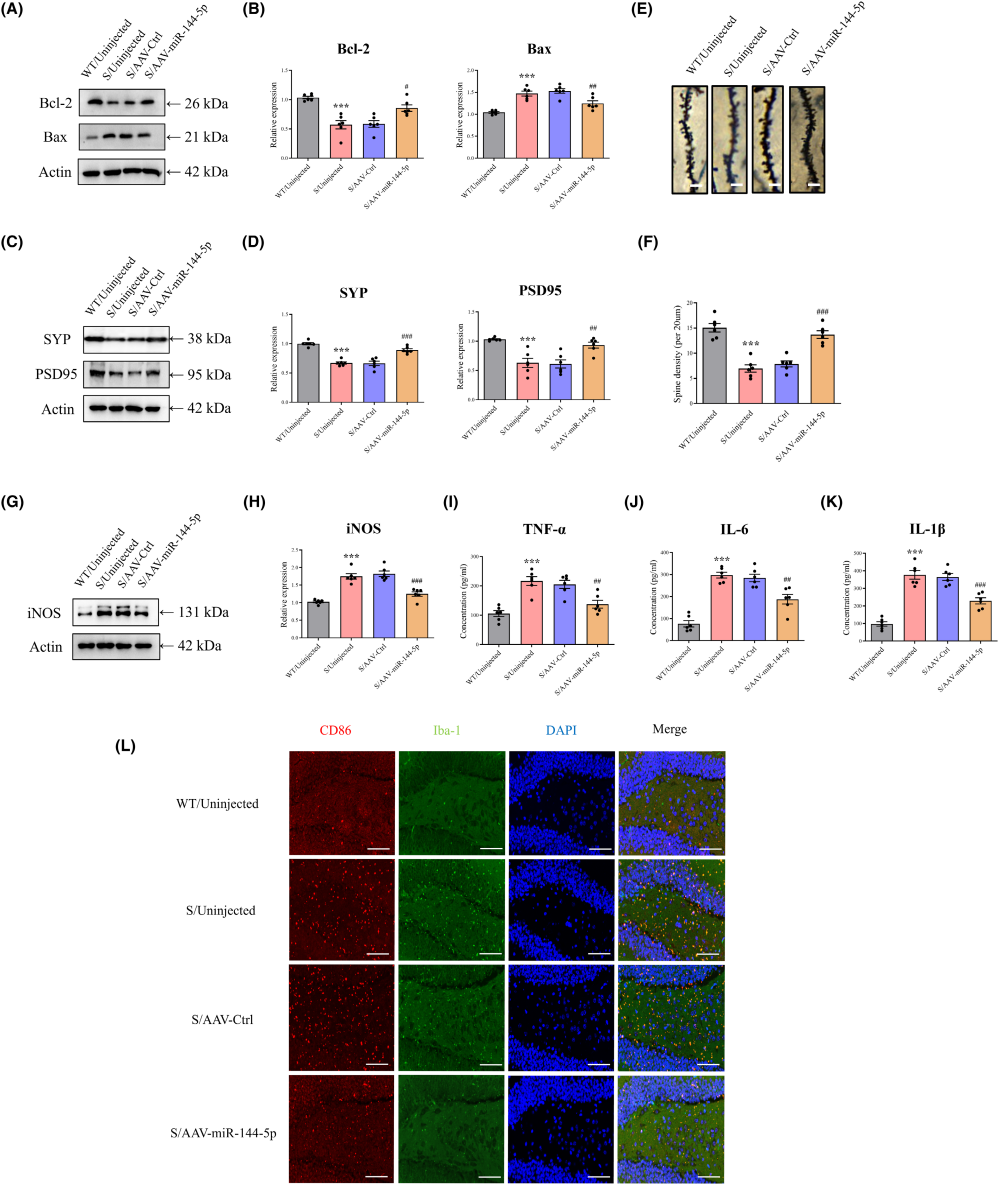
Upregulation of miR‐144‐5p rescues neuronal apoptosis and synaptic plasticity impairments and abrogates neuroinflammatory responses in CUS mice. (A) Representative bands acquired from an experiment of Bcl‐2 and Bax. (B) Overexpression of miR‐144‐5p increased levels of Bcl‐2 while preventing increase in Bax induced by CUS. *n* = 6 in each group. ***p < 0.001 versus WT/Uninjected group, ^#^ *p* < 0.05, ^##^ *p* < 0.01 versus S/AAV‐Ctrl group. (C) Representative bands acquired from an experiment of SYP and PSD95. (D) Overexpression of miR‐144‐5p reversed the reduction in the levels of SYP and PSD95 induced by CUS. *n* = 6 in each group. ***p < 0.001 versus WT/Uninjected group, ^##^ *p* < 0.01, ^###^ *p* < 0.001 versus S/AAV‐Ctrl group. (E) Representative images of the dendritic spine in Golgi staining. Scale bar: 2 μm. (F) Overexpression of miR‐144‐5p alleviated synaptic plasticity impairment induced by CUS. *n* = 6 in each group. ***p < 0.001 versus WT/Uninjected group, ^###^ *p* < 0.001 versus S/AAV‐Ctrl group. (G) Representative bands acquired from an experiment of iNOS. (H) Overexpression of miR‐144‐5p suppressed levels of iNOS. *n* = 6 mice per group. ***p < 0.001 versus WT/Uninjected group, ^###^ *p* < 0.001 versus S/AAV‐Ctrl group. Levels of cytokines such as TNF‐α (I), IL‐6 (J), and IL‐1β (K) were detected by ELISA. *n* = 6 mice per group. ***p < 0.001 versus WT/Uninjected group, ^##^ *p* < 0.01, ^###^ *p* < 0.001 versus S/AAV‐Ctrl group. (L) Representative photomicrographs of CD86/Iba1 staining. Scale bar: 50 μm.

We detected apoptosis‐related proteins to determine whether neuronal cell damage was blocked by miR‐144‐5p restoration. Decreased Bcl‐2 (anti‐apoptotic factor) and increased Bax (pro‐apoptotic factor) levels were observed in the CUS group compared to the sham group, which was mitigated by miR‐144‐5p AAV administration (Figure 4A,B). Western blot analysis revealed that the elevation of miR‐144‐5p increased the levels of neuroplasticity‐related proteins including SYP and PSD95 in CUS mice (Figure 4C,D). In addition, miR‐144‐5p upregulation alleviated the reduction in spine density caused by chronic stress, as assessed using the Golgi staining assay (Figure 4E,F).

### 3.5 miR‐144‐5p restoration inhibits microglial activation in CUS mice

Levels of pro‐inflammatory markers iNOS, TNF‐α, IL‐6, and IL‐1β were significantly elevated in CUS mice, while miR‐144‐5p AAV administration suppressed this effect (Figure 4G–K). Iba‐1 and CD86 are microglia‐specific and M1‐polarized microglial markers, respectively. To verify the function of miR‐144‐5p in neuroinflammation in mice subjected to CUS, we performed double‐labelling IF for Iba‐1+CD86. In the CUS group, more microglia were detected, and microglial expression of CD86 was increased compared to that in the sham group. Moreover, miR‐144‐5p‐AAV intervention inhibited CD86 expression in the CUS group compared to that in the control group (Figure 4L).

### 3.6 miR‐144‐5p knockdown leads to behavioral dysfunctions in normal mice

The AAV‐miR‐144‐5p‐sponge virus was injected bilaterally into the DG of normal mice (Figure S1A,B). qRT‐PCR results showed that the expression of miR‐144‐5p was downregulated by AAV‐miR‐144‐5p‐sponge virus administration (Figure S1C). Exposure to the AAV‐miR‐144‐5p‐sponge significantly decreased the degree of sucrose preference compared to that in control mice (Figure S1D). Meanwhile, AAV‐miR‐144‐5p‐sponge increased the immobility duration in the TST (Figure S1E), and FST (Figure S1F). In the OFT, AAV‐miR‐144‐5p‐sponge‐treated mice showed less interest in locomotory movement than in the control group (Figure S1G,H).

### 3.7 miR‐144‐5p knockdown increases TLR4 and PTEN expression and impairs neurogenesis in normal mice

Given that miR‐144‐5p directly regulates PTEN and TLR4 expression, we investigated TLR4 and PTEN expression in the DG of AAV‐miR‐144‐5p‐sponge‐treated mice. Levels of PTEN, TLR4, and NF‐κB p‐p65 were significantly increased, while levels of PI3K and p‐Akt were notably decreased accompanied with decreased miR‐144‐5p expression in normal mice (Figure S2A,B). miR‐144‐5p knockdown significantly downregulated the expression of DCX (Figure S2C,D) and nestin (Figure S2E,F).

### 3.8 miR‐144‐5p knockdown induces neuronal damage in normal mice

Western blot analysis revealed that miR‐144‐5p downregulation significantly increased Bax and decreased Bcl‐2 levels (Figure S3A,B). Interestingly, intervention with the AAV‐miR‐144‐5p‐sponge significantly decreased SYP and PSD95 expression compared to that in the control group (Figure S3C,D). In Golgi staining experiments, exposure to AAV‐miR‐144‐5p‐sponge significantly decreased spine density in normal mice (Figure S3E,F).

### 3.9 miR‐144‐5p knockdown induces neuroinflammatory responses in normal mice

Activation of the NF‐κB pathway is closely involved in the inflammatory response and plays a key role in the pathology of M1‐polarization in microglia.^33^ AAV‐miR‐144‐5p‐sponge treatment upregulated the expression of inflammation‐relatedindicators, including iNOS, TNF‐α, IL‐6, and IL‐1β (Figure S3G–K). Moreover, it promoted microglial M1‐polarization characterized by increased CD86 levels in Iba1‐positive cells (Figure S3L).

### 3.10 PI3K/Akt/FoxO1 signaling is involved in miR‐144‐5p deficiency‐mediated neuronal impairment

**FIGURE 5:**
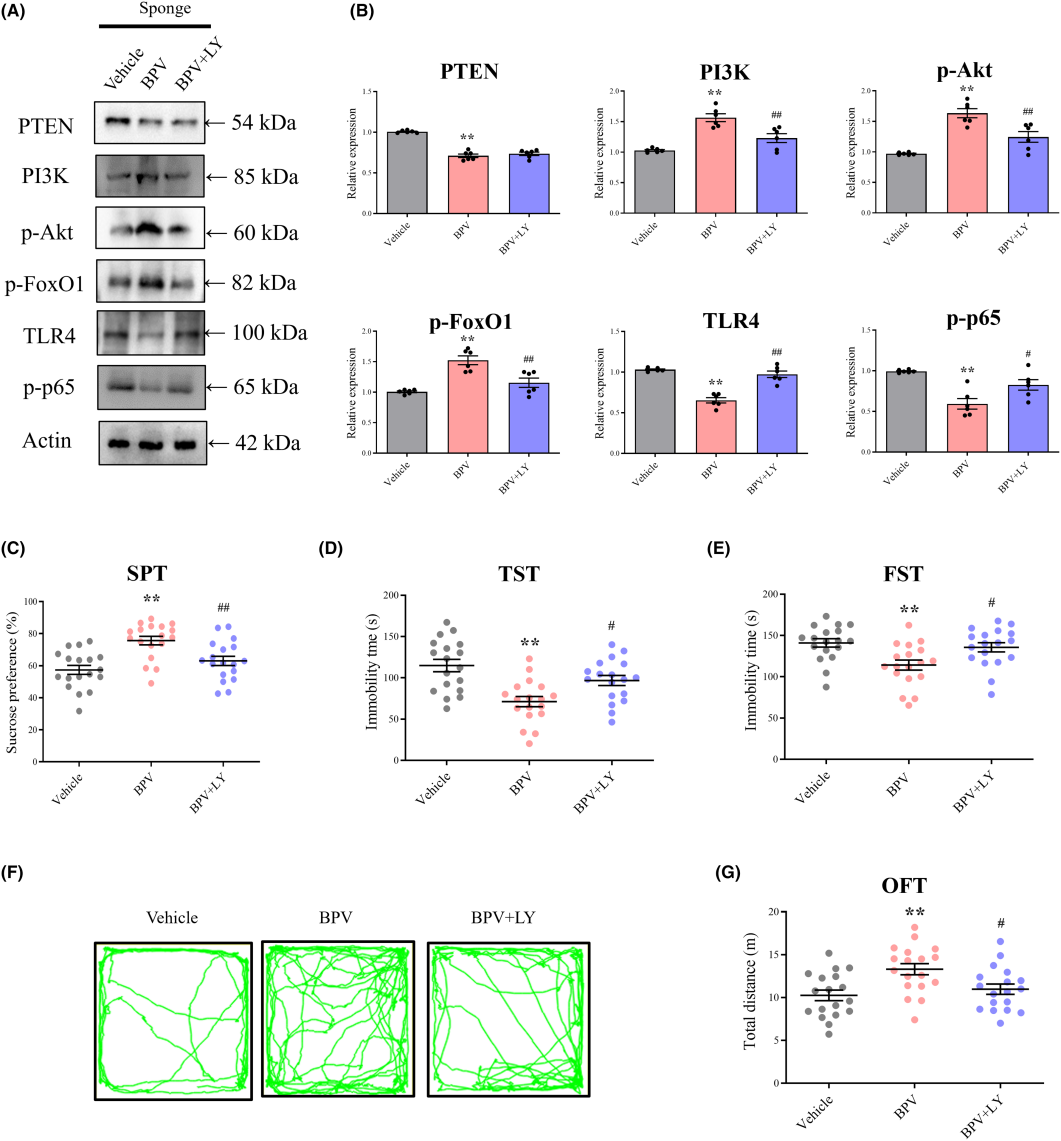
PI3K/Akt/FoxO1 signaling is involved in behavioral dysfunctions caused by miR‐144‐5p deficiency. (A) Representative bands acquired from an experiment of PTEN, PI3K, p‐Akt, p‐FoxO1, TLR4, and p‐p65. (B) Administration of bpV(pic) increased PI3K, p‐Akt, and p‐FoxO1 and decreased p‐p65 expression in mice with knockdown miR‐144‐5p. LY294002, as an inhibitor of PI3K, reversed the increase in p‐FoxO1 expression and restored the reduction in TLR4 and p‐p65 expression induced by bpV(pic) effect. *n* = 6 in each group. **p < 0.01 versus Vehicle group, ^#^ *p* < 0.05, ^##^ *p* < 0.01 versus BPV group. (C–G) Behavioral evaluation in the SPT (C), TST (D), FST (E) and OFT (F,G). *n* = 18 in each group. **p < 0.01 versus Vehicle group, ^#^ *p* < 0.05, ^##^ *p* < 0.01 versus BPV group.

FoxO1 binds to multiple enhancer‐like elements within TLR4 and exhibits transactivating activity.^34^ The PI3K/Akt/FoxO1 signaling pathway regulates TLR4 expression, thereby modifying neuroinflammation‐induced depressive behavior.^29^ We investigated the role of PTEN in regulating FoxO1‐ and TLR4‐mediated neuronal injury in mice with depression. To determine whether PTEN participates in depressive behaviors induced by miR‐144‐5p reduction after AAV‐miR‐144‐5p‐sponge intervention, we administered bpV(pic), a PTEN inhibitor. Western blot analyses showed that bpV(pic) treatment resulted in increased PI3K, p‐Akt, and p‐FoxO1 levels and reduced TLR4, NF‐κB p‐p65 levels (Figure 5A,B). However, combined treatment with LY294002, a specific blocker of PI3K, reversed the changes in p‐Akt, p‐FoxO1, TLR4, and NF‐κB p‐p65 induced by bpV(pic) in miR‐144‐5p‐knockdown mice (Figure 5A,B). Furthermore, bpV(pic) treatment significantly improved depressive symptoms caused by miR‐144‐5p deficiency, which were eliminated by the combined LY294002 treatment (Figure 5C–G). Together, these results indicate that the trigger of the TLR4 pathway is associated with elevated PI3K/Akt/FoxO1 activation by bpV(pic) treatment in miR‐144‐5p‐knockdown mice.

### 3.11 Reduced miR‐144‐5p level in serum of patients with MDD is associated with depressive symptoms

**FIGURE 6:**
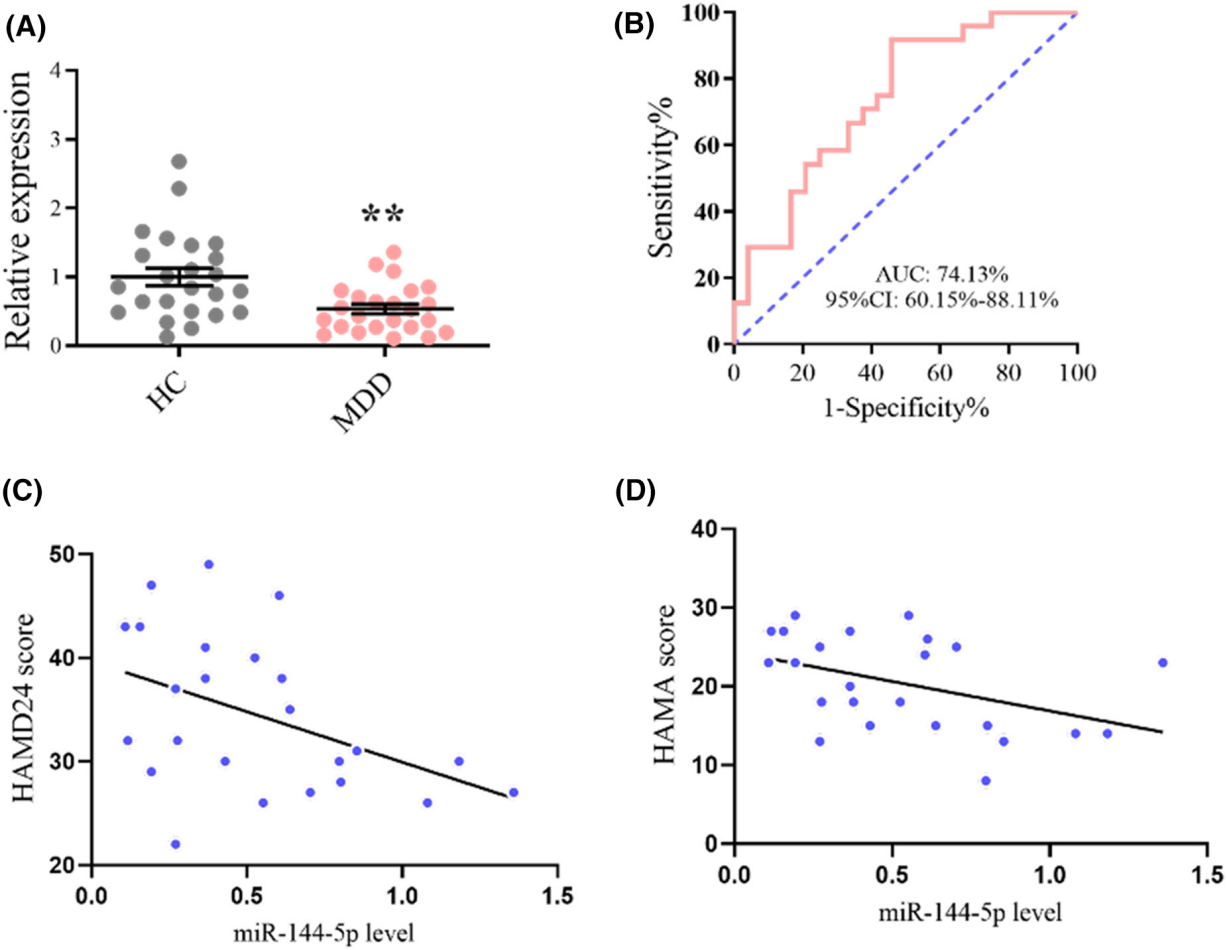
miR‐144‐5p is downregulated in MDD patients. (A) Levels of miR‐144‐5p were decreased in the serum of patients with MDD (*n* = 24) compared with HC subjects (*n* = 24). **p < 0.01 versus HC. (B) ROC analysis of miR‐144‐5p to separate MDD patients from HC subjects. (C) Correlation between miR‐144‐5p levels and HAMD‐24 score utilizing Pearson's correlation analysis. (D) Correlation between miR‐144‐5p levels and HAMA score utilizing Pearson's correlation analysis.

To investigate whether miR‐144‐5p is involved in MDD, we measured miR‐144‐5p expression in HC and patients with MDD. We observed reduced miR‐144‐5p levels in the serum of patients with MDD (Figure 6A). Receiver operating characteristic (ROC) curve analysis indicated an area under the curve (AUC) of 0.741 (95% confidence interval [CI], 0.602–0.881), with a sensitivity of 0.792 and specificity of 0.667 (Figure 6B). Similarly, miR‐144‐5p levels were lower in the serum of CUS mice than in normal mice (Figure S4). Furthermore, decreased miR‐144‐5p levels correlated with more severe depression symptoms, as measured by the HAMD‐24 (r = −0.4426, *p* = 0.03) (Figure 6C) and HAMA (r = −0.428, *p* = 0.04) (Figure 6D). The demographic and clinical characteristics of the patients are presented in Table S3. Exosomes containing miRNAs can be released by neurons and cross the blood–brain barrier for transferring biological information.^35^ Therefore, an exosome‐based blood indicator assay was performed to determine exosomal miR‐144‐5p. We first extracted serum‐derived exosomes from the clinical subjects. TEM images showed exosomes as circular double‐layered vesicles with diameters ranging from 50 to 200 nm (Figure S5A). The vesicles were approximately 100 nm in diameter, as measured using NTA (Figure S5B). Protein markers CD9 and CD63 were highly expressed in exosome samples but were not detected in the serum supernatant (Figure S5C). The levels of serum exosome‐derived miR‐144‐5p were lower in patients with MDD than those in HC, showing the trend similar to that observed in the serum samples (Figure S5D).

## 4 DISCUSSION

In the present study, we demonstrated that miR‐144‐5p was downregulated in the hippocampus of aCUS mouse model, which is consistent with its level in peripheral blood samples of CUS mice and those of patients with MDD. We also provide evidence that miR‐144‐5p in peripheral blood may serve as a biomarker for evaluating MDD severity. Furthermore, we found that miR‐144‐5p in the DG is involved in mediating CUS‐induced depressive behaviors.

The hippocampus is critically affected in depressive brain.^36^ In the present study, we investigated differentially expressed miRNAs using high‐throughput techniques in a mouse model of CUS‐induced depression and evaluated functions using bioinformatics analysis. Considering reduced miR‐144‐5p levels in mouse models of depression, we explored the relevance of this biological indicator in clinical settings. Previous studies have reported that baseline levels of miR‐144‐5p in the peripheral blood are significantly lower in depressed or anxious patients than in HC subjects.^37^, ^38^ Similarly, reports of other psychiatric disorders have indicated lower levels of blood exosome‐derived miR‐144‐5p in patients with schizophrenia than in controls.^39^, ^40^ These findings inspired us to investigate the potential function of miR‐144‐5p in MDD. Plasma miR‐144‐5p levels are negatively correlated with depression, and inflammatory proteins are negatively correlated with miR‐144‐5p levels.^38^ We identified a potential mechanism through which miR‐144‐5p regulates CNS inflammation in mice with depression. In addition, we verified miR‐144‐5p levels in different peripheral blood sample sources and explored miR‐144‐5p levels in exosomes that can cross the blood–brain barrier.

miR‐144‐5p is widely expressed in the body, including the brain.^41^ Increasing evidence indicates that miR‐144 participates in mood stabilization^42^ and stress responses.^43^ In the hippocampus, a vital brain region involved in emotion regulation, miR‐144 expression is significantly increased in response to treatment with valproate and mood stabilizers.^42^ miR‐144‐5p is involved in various diseases, including tumors, inflammatory diseases, and immune disorders; its expression is downregulated in several diseases, including Alzheimer's disease,^44^ eosinophilic esophagitis,^45^ and premature ovarian failure.^46^ Increased miR‐144 level in hypoxic cardiomyocytes improves cardiac function and remodeling. Mesenchymal stem cell exosome‐derived miR‐144 inhibits apoptosis in cardiomyocytes subjected to hypoxia.^47^ In the present study, TLR4 and PTEN were identified as two targets of miR‐144‐5p using luciferase reporter assay, and their expression decreased with miR‐144‐5p overexpression and vice versa, as verified in vivo. Consistent with our results, previous studies on brain diseases have shown that PTEN is a potential target of miR‐144‐5p.^48^, ^49^ Toll‐like receptor 2 (TLR2) regulates inflammation by acting downstream of miR‐144‐5p,^50^ and our results suggest that miR‐144‐5p downregulatesTLR4 expression.

PTEN was originally identified as a tumor suppressor that regulates cell proliferation and apoptosis by inhibiting the PI3K‐Akt signaling pathway via dephosphorylating phosphatidylinositol.^51^ PTEN signaling is common in hippocampal transcriptional features of stress models and patients with MDD.^52^ TLR4 signaling plays a pivotal role in stress‐induced neuroinflammation and MDD.^53^, ^54^ NF‐κB, a critical molecule downstream of PI3K/Akt signaling, regulates the secretion of multiple cytokines^55^ and acts as part of the TLR4 downstream signaling pathway.^56^ In our study, miR‐144‐5p restoration alleviated behavioral dysfunctions in CUS mice, and this effect was accompanied with decreased levels of PTEN and TLR4. In addition, PI3K and p‐Akt levels were increased and those of NF‐κB and p‐p65 were decreased.

There is a significant association between depression and reduced neurogenesis, and the latter can be ameliorated by antidepressant treatment.^57^ Furthermore, chronic stress leads to a reduced density of pyramidal neurons and increased apoptosis of hippocampal neurons in animal models.^23^, ^58^, ^59^ Consistent with these findings, we observed neuronal destruction due to stress indepression. PTEN deficiency upregulates differentiation and proliferation of neural stem cells.^60^ Thus, PTEN upregulation may be associated with the inhibition of PI3K/Akt signaling‐related apoptosis. Notably, neuronal cell survival and death are partially attributed to variations in PTEN levels.^61^ Recruitment of PTEN to the postsynaptic membrane leads to synaptic depression.^62^ Therapeutically, the activation of Akt by PTEN inhibition may be significant in maintaining neuroprotective properties. In addition, PTEN deficiency in retinal ganglion cells promotes axonal regeneration following optic nerve injury.^63^ Interestingly, miR‐144‐5p downregulation significantly reduced spine density accompanied with decreased levels of SYP and PSD95. Thus, our results reveal the underlying mechanisms by which miR‐144‐5p influences synaptic plasticity by targeting PTEN and TLR4.

Kéri et al. reported elevated levels of peripheral blood mononuclear cell TLR4 levels in patients with MDD; notably, as depressive symptoms improved, the levels decreased gradually compared to those of healthy controls.^64^ TLR4 protein levels increase in the hippocampus of mice subjected to chronic social stress and positively correlated with severity of depression‐like symptoms, whereas treatment with the antidepressant fluoxetine downregulated TLR4 expression and alleviated depression‐like behavior.^65^ A study on the microbiota–gut–brain axis found that Lactobacillus alleviated depression‐like behavior in mice via TLR4 signaling.^66^PTEN inhibition alleviates neuroinflammation by regulating PI3K/Akt signaling.^67^ PTEN/PI3K/Akt signaling regulates the NF‐κB pathway and impacts the release of inflammatory cytokines.^68^ NF‐κB signaling pathway activation is involved in the development of depression.^69^ In ischemic stroke, miR‐144 suppresses neuroinflammation and exerts neuroprotective effects through the PTEN/Akt pathway.^70^ Consistent with these findings, our results show that miR‐144‐5p attenuates neuroinflammation in depression by regulating PTEN signaling. Inflammatory protein expression is reduced in patients with anxiety, depression, or stress and adjustment disorders after psychotherapy, and these variations are influenced by miR‐144‐5p.^38^ In the study, we demonstrated that miR‐144‐5p exerts anti‐inflammatory effects in patients with MDD.

To clarify whether TLR4 and PTEN/PI3K/Akt signaling are involved in stress vulnerability and targeted by miR‐144‐5p, we first examined the expression of TLR4 and PTEN and determined that both were increased following miR‐144‐5p downregulation. The PTEN‐mediated Akt/β‐catenin/FoxO1 pathway regulates TLR4‐mediated innate immune responses.^71^ Multiple isoforms of FoxO exist in the CNS, but FoxO1 is mainly found in the striatum and hippocampus.^72^ A previous study reported that FoxO1‐knockout mice show reduced anxiolytic behavior.^73^ Furthermore, FoxO1 expression increases during inflammatory processes.^74^ Taken together, our results suggest that PI3K/Akt/FoxO1 signaling may be involved in the pathophysiology of depression.

This study has several limitations. The present study did not include a definitive exploration of the brain cell types from which miR‐144‐5p is derived. Extracellular vesicles have been used for the targeted delivery of exogenous nucleic acid drugs in vivo, and it is necessary to further confirm the underlying mechanisms of miR‐144‐5p in MDD based on different drug delivery platforms in future studies.

## 5 CONCLUSION

miR‐144‐5p plays a vital role in regulating neuronal abnormalities in depression. Our findings provide translational evidence that miR‐144‐5p is a new potential therapeutic target for MDD.

## AUTHOR CONTRIBUTIONS

HL, ZR, and XW contributed to the study design. XW, ZS, PW, and CW performed the depression model and behavioral tests. XL, QZ, BL, ZL, and YY performed clinical blood sample collection and scale assessment. XW performed TEM experiments. XW YZ, and XL performed drug administration intervention, molecular biology experiments, and analysis. XW drafted the manuscript with subsequent iterations refined by HL and ZR.

## FUNDING INFORMATION

This research was supported by Anhui Provincial Natural Science Foundation (2108085MH275), the National Natural Science Foundation of China (82071401), Scientific Research Project of Education Department of Anhui Province (YJS20210278), and the National Clinical Key Specialty Project Foundation (CN).

## CONFLICT OF INTEREST STATEMENT

The authors declare no conflicts of interest.

## Supporting information


Appendix S1

## Data Availability

The data that support the findings of this study are available from the corresponding author upon reasonable request.
