# Supplementary material for: Clinical and preclinical evaluation of miR‐144‐5p as a key target for major depressive disorder
Source: CNS Neurosci Ther. 2023 Jun 12;29(11):3598–611. doi: 10.1111/cns.14291 (PMC10580367; doi:10.1111/cns.14291)
Supplement: Supplementary file 1 — Appendix S1 [file CNS-29-3598-s001.docx]

**Supplemental figure**

**Fig. S1** Knockdown of miR-144-5p in the DG leads to depression-like phenotypes.

**A** Experimental paradigm for AAV-miR-144-5p-sponge intervention. **B** Schematics of AAV-miR-144-5p-sponge. **C** The infection efficiency of AAV-miR-144-5p-sponge. n = 6 in each group. ^##^*p* < 0.01 vs. AAV-Ctrl group. **D-H** Behavioral effects of expressed AAV-miR-144-5p-sponge in the DG. Mice with knockdown miR-144-5p displayed depressive-like behaviors as measured by the SPT (**D**), TST (**E**), FST (**F**) and OFT (**G, H**). n = 24 in each group. ^##^*p* < 0.01, ^###^*p* < 0.001 vs. AAV-Ctrl group.


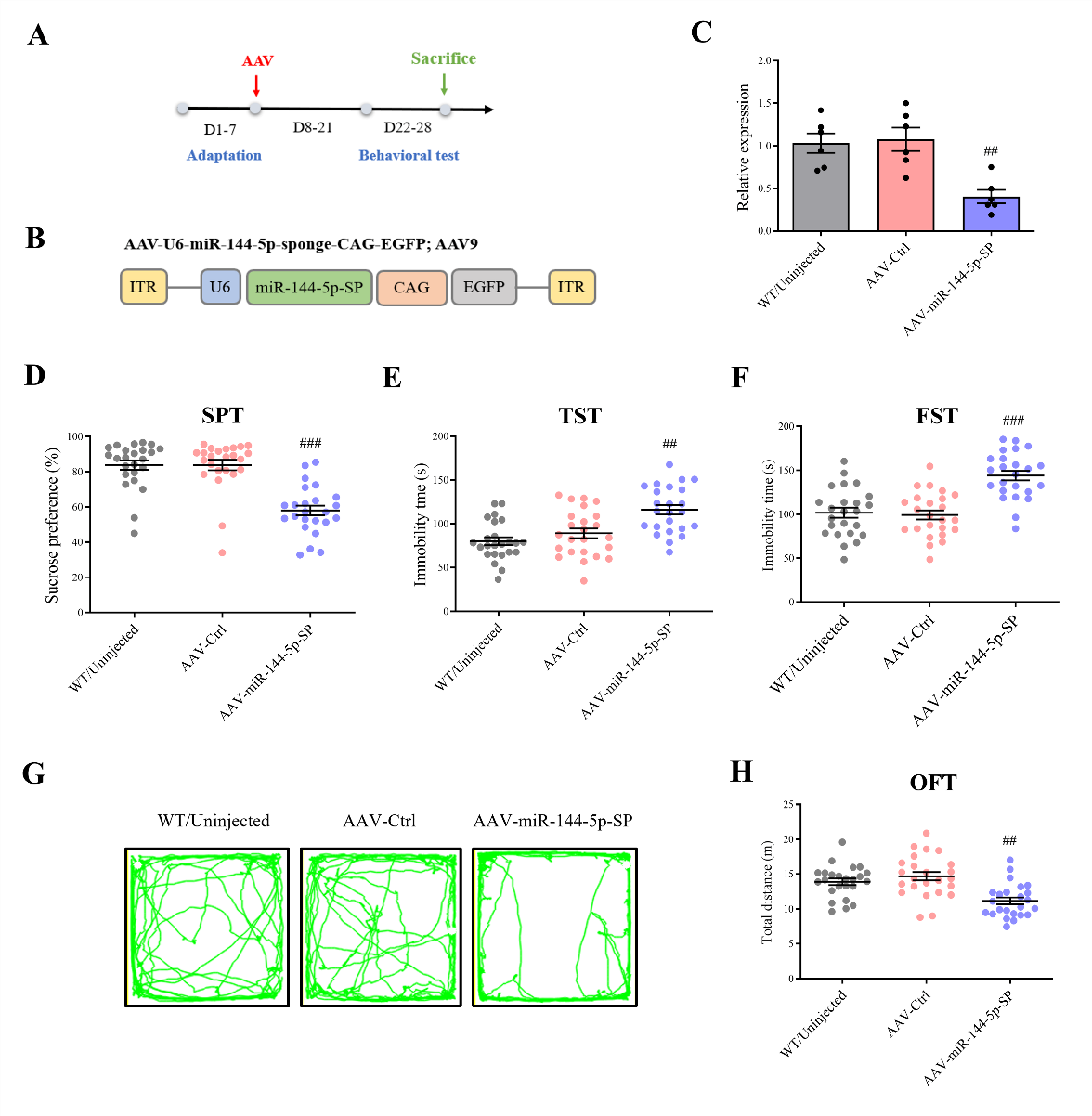


**Fig. S2** Knockdown of miR-144-5p suppresses neuronal neurogenesis in normal mice.

**A** Representative bands acquired from an experiment of TLR4, PTEN, PI3K, p-Akt, and p-p65. **B** Knockdown of miR-144-5p increased levels of TLR4, PTEN and p-p65 while decreasing levels of PI3K and p-Akt. n = 6 in each group. ^###^*p* < 0.001 vs. AAV-Ctrl group. **C-F** Representative photomicrographs showing DCX^+^ and Nestin^+^ cells in the DG. Scale bar: 50 µm. n = 6 in each group. ^##^*p* < 0.01 vs. AAV-Ctrl group.


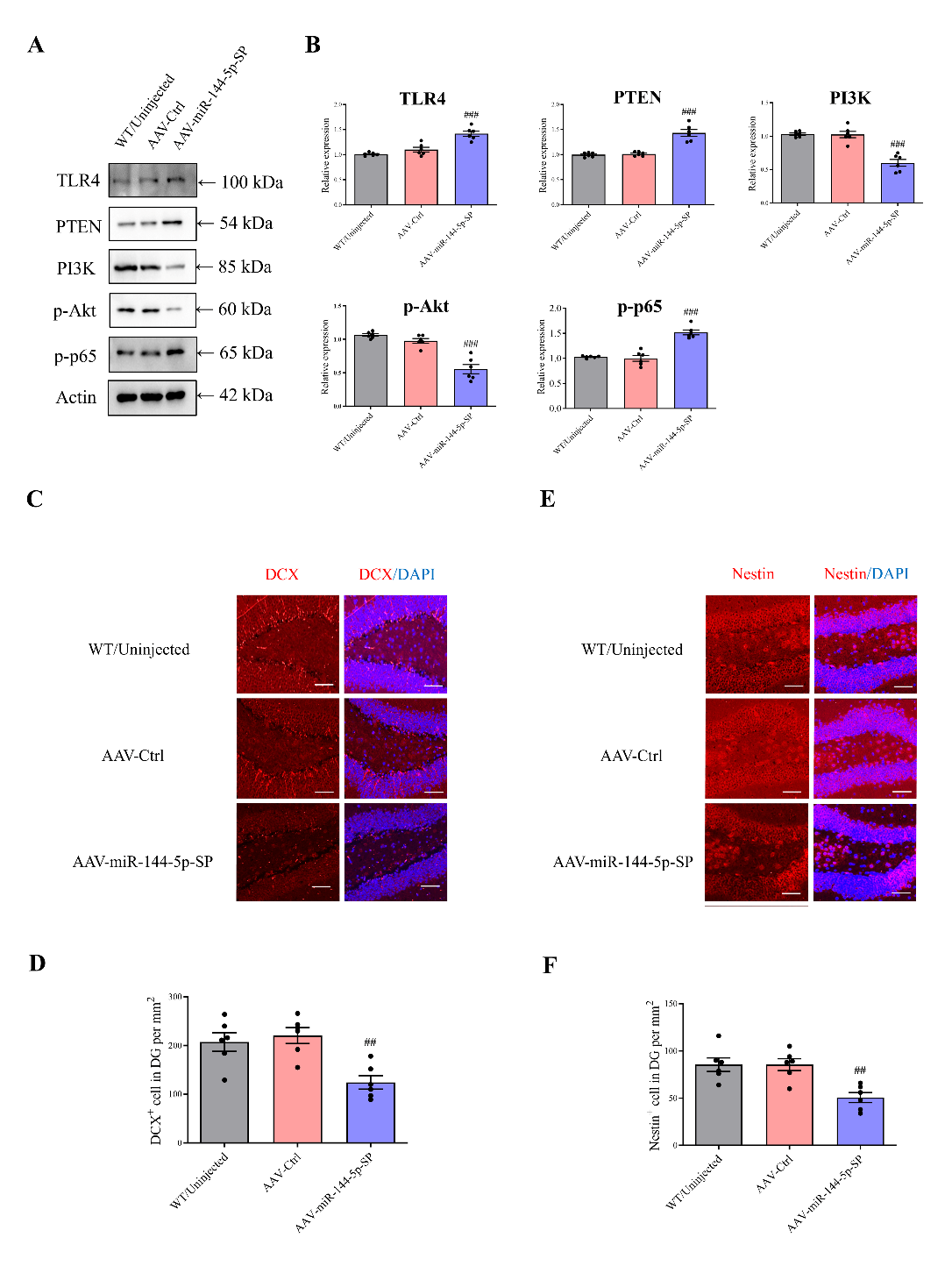


**Fig. S3** Knockdown of miR-144-5p induces neuronal apoptosis, synaptic plasticity impairments and neuroinflammation in normal mice.

**A** Representative bands acquired from an experiment of Bcl-2 and Bax. **B** Knockdown of miR-144-5p enhanced Bax while suppressing Bcl-2 expression. n = 6 in each group. ^###^*p* < 0.001 vs. AAV-Ctrl group. **C** Representative bands acquired from an experiment of SYP and PSD95. **D** Knockdown of miR-144-5p reduced the immunocontent of SYP and PSD95. n = 6 in each group. ^###^*p* < 0.001 vs. AAV-Ctrl group. **E** Representative images of the dendritic spine in Golgi staining. Scale bar: 2 µm. n = 6 in each group. **F** Knockdown of miR-144-5p reduced spine density in the DG. n = 6 in each group. ^###^*p* < 0.001 vs. AAV-Ctrl group. **G** Representative bands acquired from an experiment of iNOS. **H** Knock-down of miR-144-5p increased expression of iNOS. n = 6 mice per group. ^###^*p* < 0.001 vs. AAV-Ctrl group. Levels of cytokines such as TNF-α (**I**), IL-6 (**J**), and IL-1β (**K**) were detected by ELISA. n = 6 mice per group. ^##^*p* < 0.01, ^###^*p* < 0.001 vs. AAV-Ctrl group. **L** Representative photomicrographs of CD86/Iba1 staining. Scale bar: 50 μm.


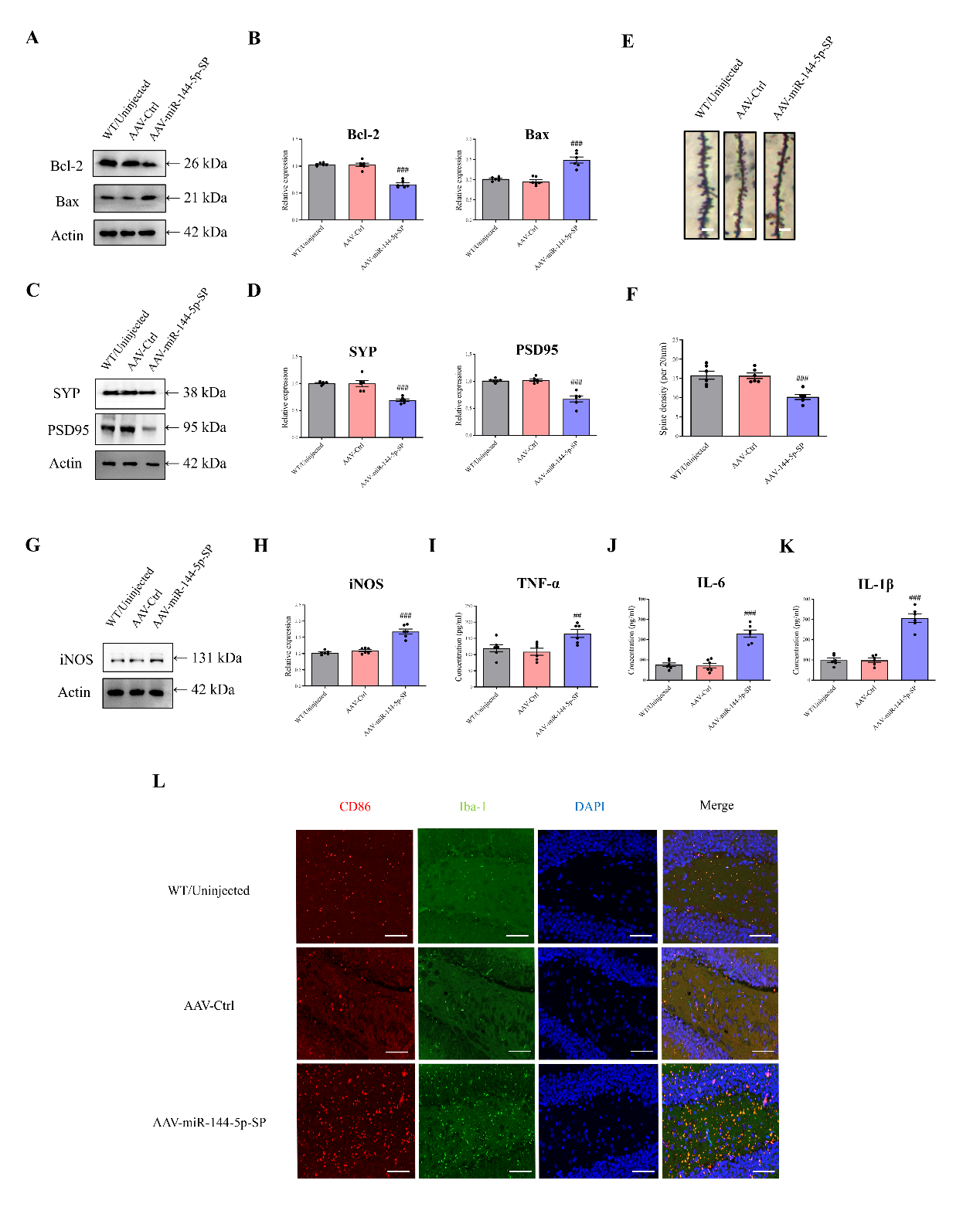


**Fig. S4** miR-144-5p is downregulated in the serum of CUS mice. n = 12 in each group. ***p* < 0.01 vs. Ctrl.


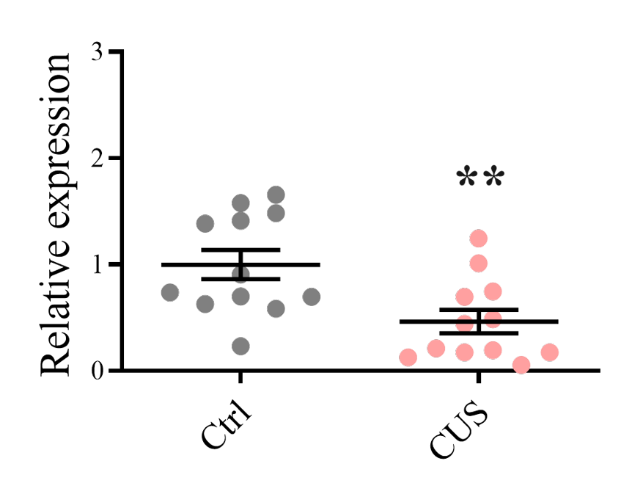


**Fig. S5** miR-144-5p is downregulated in serum-derived exosomes of MDD patients.

**A-C** Characteristics of exosomes was profiled with TEM (**A**), NTA (**B**) and western blotting (**C**). Scale bar: 200 µm. **D** Levels of miR-144-5p were reduced in serum-derived exosomes of MDD patients (n = 24) compared with HC subjects (n = 24). ***p* < 0.01 vs. HC.


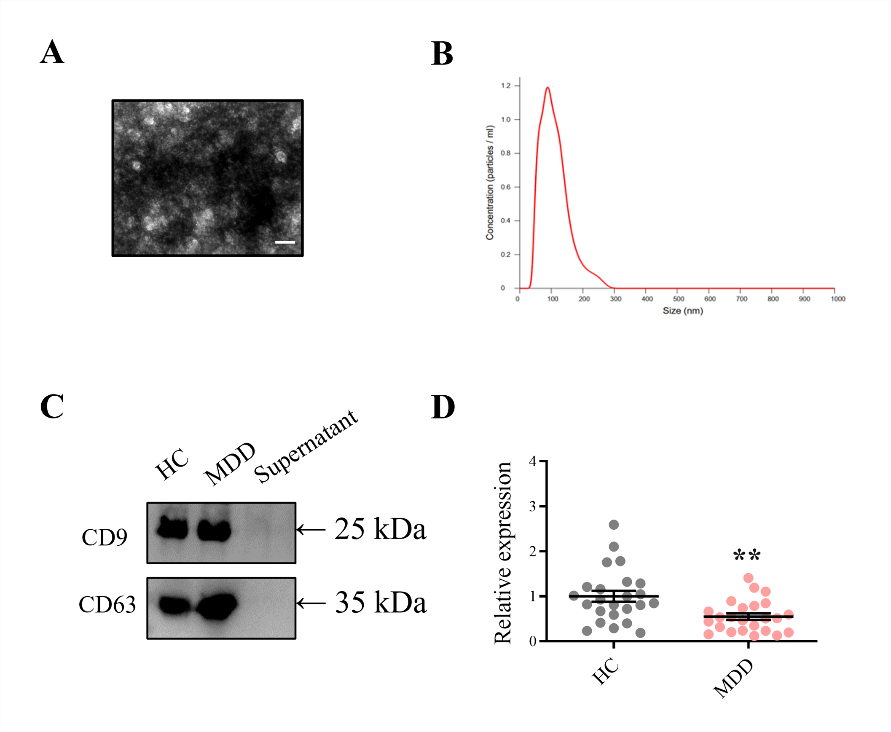


**Supplemental table**

**Table S1** The information of Antibodies and ELISA Kits.

| **Antibody** | **Vendor** | **Catalog Number** |
| --- | --- | --- |
| PTEN | Cell Signaling Technology | #9188 |
| TLR4 | Cell Signaling Technology | #14358 |
| PI3K | Cell Signaling Technology | #4257 |
| p-Akt | Cell Signaling Technology | #4060 |
| NF-κB p-p65 | Cell Signaling Technology | #3033S |
| Bcl2 | Proteintech Group | 26593-1-AP |
| Bax | Proteintech Group | 50599-2-Ig |
| SYP | Proteintech Group | 17785-1-AP |
| PSD95 | Proteintech Group | 20665-1-AP |
| iNOS | Proteintech Group | 18985-1-AP |
| β-actin | Proteintech Group | 66009-1-Ig |
| p-FoxO1 | Cell Signaling Technology | #9464 |
| Nestin | Proteintech Group | 19483-1-AP |
| DCX | Proteintech Group | 13925-1-AP |
| CD86 | ABclonal | A16805 |
| Iba-1 | Servicebio | GB12105 |
| Cy3 | Servicebio | GB21303 |
| Cy5 | Servicebio | GB27301 |
| DAPI | Beyotime | C1002 |
| CD9 | Proteintech Group | 20597-1-AP |
| CD63 | Immunoway | YT5525 |
| Mouse TNF-α | Elabscience | E-EL-M3063 |
| Mouse IL-6 | Elabscience | E-EL-M0044c |
| Mouse IL-1β | Elabscience | E-EL-M0037c |

**Table S2** Up-regulated and down-regulated expressed miRNAs ranked by *p*-value.

| miRNA_id | log2FoldChange | p-Value | Regulation |
| --- | --- | --- | --- |
| mmu-miR-10a-5p | -0.854168953 | 4.09E-05 | Down |
| mmu-miR-126b-5p | -0.6026958 | 0.008586 | Down |
| mmu-miR-143-3p | -0.798021553 | 0.00496 | Down |
| mmu-miR-144-3p | -0.857251414 | 0.015334 | Down |
| mmu-miR-144-5p | -0.867805956 | 0.012889 | Down |
| mmu-miR-199a-3p | -0.631385969 | 0.004502 | Down |
| mmu-miR-199a-5p | -0.700427621 | 0.003711 | Down |
| mmu-miR-211-5p | -2.350826092 | 2.78E-06 | Down |
| mmu-miR-214-5p | -1.043898635 | 0.027232 | Down |
| mmu-miR-30f | -0.66160081 | 0.006276 | Down |
| mmu-miR-33-3p | 1.056335221 | 0.042225 | Up |
| mmu-miR-363-3p | -1.082705224 | 0.005614 | Down |
| mmu-miR-378d | -0.626781132 | 0.002498 | Down |
| mmu-miR-451a | -0.819011185 | 0.00232 | Down |
| mmu-miR-466b-3p | 2.094950663 | 0.029048 | Up |
| mmu-miR-5106 | -5.421450284 | 0.001081 | Down |
| mmu-miR-5134-3p | 3.474873134 | 0.047276 | Up |

**Table S3** Demographic and Clinical Characteristics of patients.

| **Characteristic** | **MDD (n=24)** | **HC (n=24)** |
| --- | --- | --- |
| Age (years) | 31.9 ± 13.8 | 31.6 ± 12.5 |
| Gender (male/female) | 7/17 | 11/13 |
| HAMD score | 34.5 ± 7.4 | - |
| HAMA score | 20.37 ± 5.9 | - |
